# 

**Published:** 1881-05

**Authors:** 


					﻿MAY, 1881.
TWENTY-EIGHTH YEAR.
IIAELS

OF
HEALTH
E. H, GIBBS, A.M., M.D., Editor,
No. 141 EIGHTH STREET (Near Broadway), NEW YORK.
TERMS: $2 a Year, or $1 for Six Montis. Single nnmier, 20 ctsJ
				

